# Contact sites between host organelles and pathogens: boon or bane?

**DOI:** 10.1128/msphere.00448-23

**Published:** 2023-10-18

**Authors:** Chahat Mehra, Lena Pernas

**Affiliations:** 1 Max Planck Institute for Biology of Ageing, Cologne, Germany; 2 Cologne Excellence Cluster on Cellular Stress Responses in Ageing-Associated Diseases (CECAD), University of Cologne, Cologne, Germany; 3 Department of Microbiology, Immunology and Molecular Genetics, University of California, Los Angeles, California, USA; University at Buffalo, Buffalo, New York, USA

**Keywords:** membrane, membrane contact sites, mitochondria, endoplasmic reticulum, *Toxoplasma gondii*, pathogens

## Abstract

A microbe and its host are in constant communication. An emerging platform for direct communication is the membrane contact sites that form between several pathogens and host organelles. Here, we review our progress on the molecular mechanisms underlying contact sites between host mitochondria and the human parasite *Toxoplasma gondii*. We discuss open questions regarding their function during infection as well as those formed between the host endoplasmic reticulum and *Toxoplasma*.

## INTRODUCTION

A microbe and its host are in constant communication. This communication can be indirect, for example, through host innate immune receptors that detect microbial molecules or occur directly through pathogen effectors that interact with host proteins. An emerging platform for direct communication is membrane contact sites (MCSs), a term used to define regions of membrane apposition between organelles in a 10–30 nm range. MCSs form between several pathogens and host organelles (1, 2). How MCSs are formed, the extent to which they mediate host-pathogen communication, and the role they play during infection remain open questions.

Host-pathogen contact sites were first described in cells infected with *Toxoplasma gondii* (*Toxoplasma*)*,* an obligate intracellular parasite of warm-blooded hosts including humans (3). Following invasion, *Toxoplasma* resides in a compartment termed the parasitophorous vacuole (PV) and becomes surrounded by host mitochondria, termed host mitochondrial association (HMA). A hallmark of HMA is the contact site formed between the PV membrane (PVM) and the outer mitochondrial membrane (OMM) (4, 5). Since its discovery in *Toxoplasma*-infected cells, HMA has been found to occur during infection with evolutionary diverse pathogens, such as the eukaryotic parasite *Microsporidia*, and the prokaryotic pathogens *Legionella pneumophila* and *Chlamydia psittaci* (6, 7).

The interaction between *Toxoplasma* and mitochondria was initially speculated to result from the physical constraint imposed by the growing PV (5). Yet, similarly sized vacuoles such as those formed by the related apicomplexan *Neospora caninum* or the bacteria *Coxiella burnetti* do not exhibit HMA (5, 8). More comprehensive analyses revealed that HMA persists throughout infection as well as in cells treated with ultraviolet light, toxoplasmicidal agents, or following cell lysis (5, 9). Collectively, these studies instead supported a specific interaction between the *Toxoplasma* PV and host mitochondria that was analogous to the protein-mediated contact sites formed between host organelles (1).

The discovery that HMA differed between the three predominant strains of *Toxoplasma*—types I and III are HMA-positive unlike type II—enabled the identification of the parasite effector mitochondrial association factor 1 (TgMAF1) that is required and sufficient for HMA (10). With the parasite mediator of HMA in hand, the host factor became discoverable, and subsequent work identified the host OMM protein TOM70 as the TgMAF1 “receptor” (11, 12). Furthermore, TOM70 was shown to directly bind TgMAF1 *in vitro* (12). As with TgMAF1, the loss of TOM70 abrogates HMA (11, 12). Thus, host mitochondria and *Toxoplasma* form trans-kingdom contact sites mediated by single protein tethers.

Despite the information about the host and parasite tethering molecules, the role of mitochondrion-*Toxoplasma* contact sites during infection remains enigmatic. Most theories advocate parasite-motivated functions. Early observations that vacuoles containing live parasites were associated with mitochondria fueled hypotheses that parasites used mitochondria to shield the vacuole from lysosomes and thus render them non-fusogenic (4). However, this is probably not the case as type II parasites that do not tether mitochondria due to suppressed expression of TgMAF1 also do not fuse with lysosomes (10). Another possibility is that *Toxoplasma* benefits from host mitochondrial ATP. Studies investigating stress-induced differentiation reported that *Toxoplasma* grew more slowly in cells deficient for mtDNA and thus mitochondrial ATP synthesis (13). However, other work reported similar parasite growth in respiration-competent and respiration-deficient host cells (14). Therefore, whether HMA confers any initial benefit to *Toxoplasma* remains to be determined.

With the emergence of the field of mitochondrial dynamics, it became relevant to view HMA from a host lens. Do mitochondria perhaps play a defensive role on behalf of the host cell? Work addressing this alternative perspective found that mitochondria serve as nutrient competitors to *Toxoplasma*. Although *Toxoplasma* exploits host cell lipophagy to access host fatty acids (FAs) it needs to grow, mitochondria restrict the parasite’s access to FAs by fusing to elongate, which increases mitochondrial uptake of FAs. A deficiency in mitochondrial fusion or mitochondria FA uptake led to increased parasite growth (15). These findings suggest that mitochondria mount a metabolic defense against the parasite at early stages of infection and also reify a host motive for mitochondrial contact with the pathogen.

Based on Van Valen’s red queen hypothesis (states that species must be in constant change to continue in the same place and thus antagonistic interactions between species lead to coevolution), one may expect a pathogen counter response (16). The question therefore arises as follows: how do parasites counter mitochondrial nutrient defense? In recent work, we discovered that mitochondria in contact with the *Toxoplasma* PV shed large structures positive for outer mitochondrial membrane termed SPOTs (12). This fascinating mitochondria-derived structure is enriched with proteins of the OMM including those required for mitochondrial nutrient defense but not proteins of the inner mitochondrial membrane (IMM) or matrix. SPOTs are readily visible budding off the OMM at contact sites, which led us to ask if the molecular tethers TgMAF1 and TOM70 mediated their formation. Indeed, the loss of either TgMAF1 or TOM70 abrogated SPOT formation (12). Although the loss of MAF1 did not affect the growth of *Toxoplasma*, the loss of TOM70 decreased parasite proliferation in a MAF1-dependent manner. Collectively, these findings suggest that *Toxoplasma* and mitochondria contact sites are the site of a molecular arms race.

Despite these recent discoveries, numerous questions about this trans-kingdom contact site remain unanswered. How do these contact sites evolve from an apparent host defense (mitochondrial nutrient competition) to a parasite counter defense (*Toxoplasma*-induced OMM shedding)? Who approaches whom? Unlike the parasites contained within a vacuole, mitochondria strategically traffic within a cell. For example, mitochondria migrate to the leading edge of cancer cells to provide the ATP required for migration (12). Thus, mitochondria may sense the presence of the parasite through as of yet unidentified cues and traffic to the PV of *Toxoplasma* to restrict its access to FAs. Another potential host defense that may be mediated at contact sites includes the release of molecules that inhibit microbial growth such as mitochondrial reactive oxygen species (13, 14).

Alternatively, one can envision several reasons for why *Toxoplasma* may benefit from proximity to host mitochondria. *Toxoplasma* scavenges lipoate (10) and cardiolipin from its host, which are synthesized in host mitochondria (11). Indeed, *Toxoplasma* but not *Neospora* recruits around its vacuole host microtubules along which mitochondria traffic (17). Thus, it is tempting to speculate that *Toxoplasma* coopts host microtubules to reroute mitochondria to the parasite vacuole. In line with this, depolymerized microtubules reduced *Toxoplasma*-mitochondria contact site formation (5).

However, HMA is not essential for the host or pathogen survival as it only occurs during infection with certain *Toxoplasma* strains as aforementioned. Exogenous expression of TgMAF1 in type II parasites confers a competitive growth advantage *in vivo*, but the loss of MAF1 does not affect parasite growth *in vitro*, except in cells deficient for TOM70 (12, 18). Thus, despite knowledge of the factors required for mitochondria-*Toxoplasma* contact sites, the question remains as follows: what is the role of these contact sites in the outcome of infection? Are there host-pathogen interactions that are redundant with HMA?

The coating of the *Toxoplasma* PV by the endoplasmic reticulum (ER), hereafter referred to as host ER association (hERa), was observed at the same time as mitochondria (4, 5). Preliminary characterizations of hERa demonstrate that as with HMA, hERa stays intact even after aerolysin-induced ER vacuolization or cellular enucleation (19). Unlike HMA, hERa occurs early after *Toxoplasma* invasion but decreases as infection progresses (3). Decades later, the mechanism underlying hERa remains unknown. What is the significance of *Toxoplasma*-ER MCSs? Myriad possibilities emerge if we consider both the host and parasite perspectives. First, *Toxoplasma* is constantly replicating within a cell and so requires lipids for membrane biogenesis as well as PV growth. This high demand for lipids could be met in part by scavenging lipids at contact sites with the host ER. Such a host-pathogen interaction is not unprecedented. The MCS between the *Chlamydia trachomatis* vacuole and host ER mediates ceramide transfer (20), enabling *Chlamydia* to exploit hERa for host lipids. Second, *Toxoplasma* could redirect ER-Golgi trafficking to acquire nutrients that help maintain the parasite vacuolar niche. This has been reported for the bacterial pathogen *Brucella abortus. B. abortus* releases the effector BspB that interacts with the conserved oligomeric Golgi (COG) complex and redirects membrane vesicles to its vacuole (21). Depletion of COG components impairs BspB dependent-*Brucella* vacuole biogenesis and bacterial replication (21).

Alternatively, hERa may support pro-host functions by facilitating host detection of microbial molecules. For example, detection of *Toxoplasma* DNA at hERA could trigger cGAS, thereby activating the ER-membrane protein stimulator of interferon (IFN) genes (STING) and innate immune signaling (22). ER-microbe contacts may activate the unfolded protein response (UPR) that initially restricts the growth of several pathogens such as *Simkania negevensi*, which tightly associates with host ER (23). As is the case with mitochondria, ER-parasite interaction may promote parasite viability and proliferation, or enable host recognition and/or activation of innate immune defenses. The identification of the host and parasite factors that mediate host ER-pathogen contact sites will enable a better understanding of the physiological relevance of this interaction.

Does the conversation end at the mitochondria and ER? Beyond the ER and mitochondria, host microtubules, the microtubule organizing center, endolysosomes, Golgi and Golgi-derived vesicles, and lipid droplets also congregate around the *Toxoplasma* PV. Do these organelles also form contact sites with the PV and if so, how and why are they formed? Is there a conserved mechanism by which different parasite effector proteins target and tether host organelles? Is the interaction with different organelles of a competitive or cooperative nature? How does the parasite decide which organelles it wants to associate with and for how long?

The progress we have made in defining the players and the biological significance of mitochondria-*Toxoplasma* MCSs is only the tip of the iceberg. Dissecting the mechanisms that regulate the formation and function of host-pathogen contact sites will allow us to better understand their coevolution and how they are adapted to promote either microbial or host survival and defenses.
